# Insights and preventive approaches of rod erosion in the occipital bone after complex posterior cervical spine surgery for destructive spondyloarthropathy: A case report

**DOI:** 10.1097/MD.0000000000037143

**Published:** 2024-02-16

**Authors:** Hirohito Hirata, Tadatsugu Morimoto, Masatsugu Tsukamoto, Takaomi Kobayashi, Tomohito Yoshihara, Yu Toda, Masaaki Mawatari

**Affiliations:** a Department of Orthopaedic Surgery, Faculty of Medicine, Saga University, Nabeshima, Saga, Japan

**Keywords:** destructive spondyloarthropathy, occipital bone erosion, skull base erosion

## Abstract

**Rationale:**

Complications of rod migration into the occipital bone after upper cervical fusion are very rare. No other cases have been reported, especially when associated with destructive spondyloarthropathy (DSA). The purpose of this case report is to remind clinicians of the risk of rod migration in cervical spine surgery in patients with DSA and to provide information on its causes, countermeasures, and treatment.

**Patient concern:**

This case report presents the clinical course of a 61-year-old female patient with chronic kidney disease that required hemodialysis.

**Diagnosis, intervention, outcomes:**

The patient was diagnosed DSA involving the cervical spine. Initial treatment involved a halo vest, followed by anterior cervical corpectomy and fusion spanning from C5 to Th1. However, subsequent complications, including C5 fractures, kyphotic cervical alignment, and rod migration into the occipital bone, lead to multistage surgical interventions. This case highlights the challenges in managing DSA, the significance of optimal fixation strategies, and the importance of accounting for potential alignment changes.

**Conclusion:**

The effective management of occipital bone erosion after posterior cervical spine surgery for destructive spondyloarthropathy necessitates meticulous fixation planning, proactive rod length adjustment, preoperative assessment of the occipital position, and consideration of the compensatory upper cervical range of motion to prevent migration-related issues.

## 1. Introduction

Destructive spondyloarthropathy (DSA) is progressive vertebral body destruction observed in patients undergoing dialysis and is often attributed to the deposition of beta-2 microglobulin amyloidosis.^[1,2]^ The rapid progression of spinal destruction associated with this condition poses a significant challenge for spinal surgeons. Surgical interventions in patients undergoing dialysis not only carry risks of wound infection and cardiovascular complications during the perioperative period but also have a higher likelihood of implant loosening and adjacent segment pathology.^[3]^ Determining the surgical approach and indications for such cases is challenging.

Herein, we present multiple operative failures due to our insufficient consideration of DSA.

## 2. Case presentation

**Figure 1.**
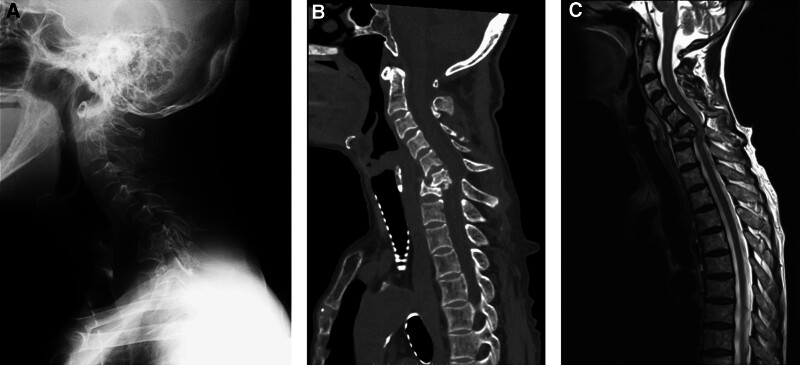
A 61-year-old female with chronic kidney disease (CKD) presented with destructive spondyloarthropathy (DSA) on C6 and C7. Lateral views of (A) the X-ray, (B) computed tomography (CT), and (C) Magnetic resonance imaging (MRI) T2 weighted image (T2WI).

**Figure 2.**
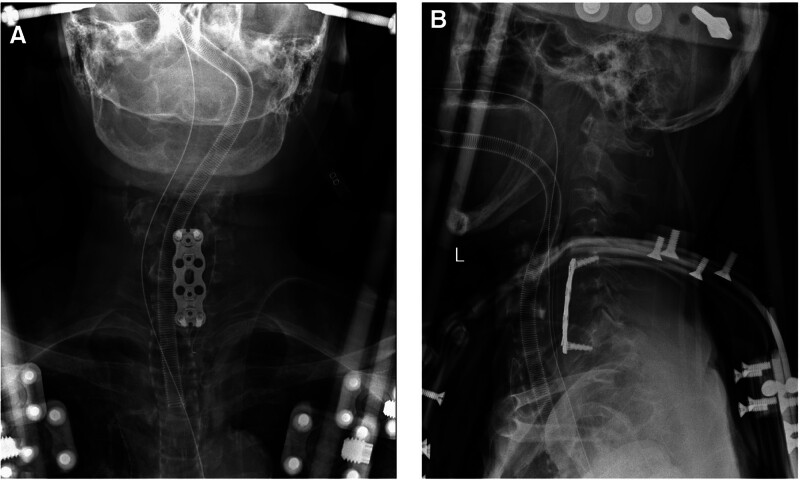
Cervical spine X-ray postanterior cervical corpectomy and fusion (ACCF). (A) the anteroposterior view; (B) the lateral view.

**Figure 3.**
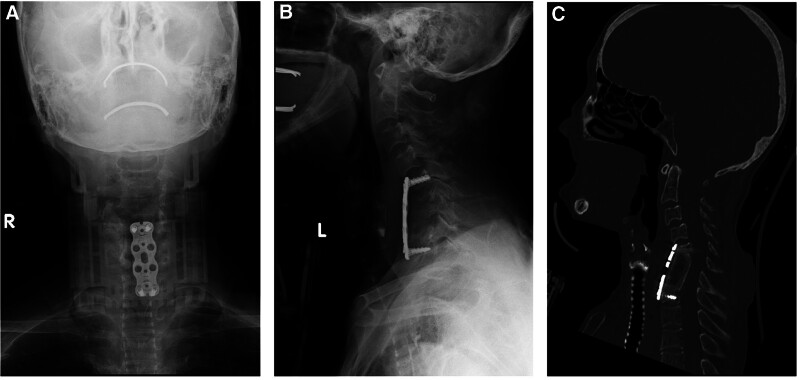
Cervical spine X-ray and CT after 1 week of anterior cervical corpectomy and fusion (ACCF). (A) The anteroposterior view; (B, C) the lateral view.

**Figure 4.**
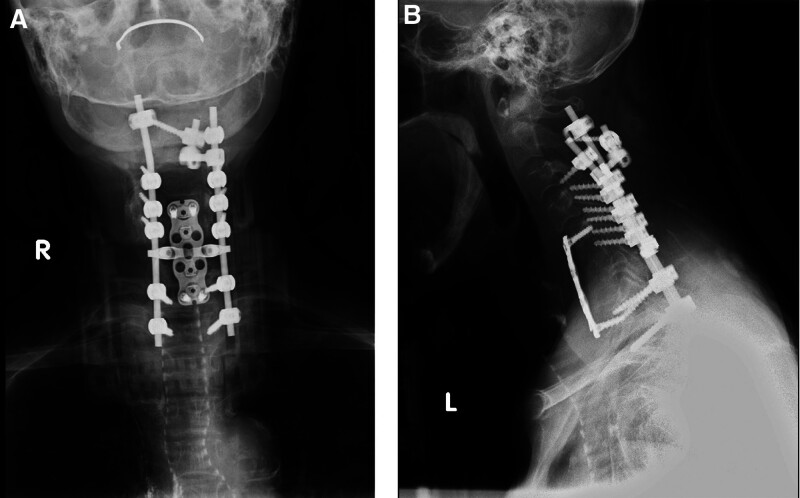
Cervical spine X-ray after posterior operation performed. (A) The anteroposterior view; (B) the lateral view.

**Figure 5.**
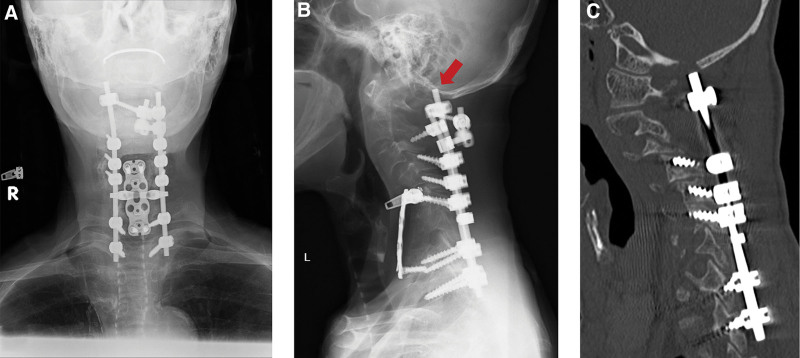
Cervical spine X-ray and computed tomography (CT) after 6 months of posterior fusion. (A) The anteroposterior view. The arrow shows the occipital bone erosion; (B, C) the lateral view.

**Figure 6.**
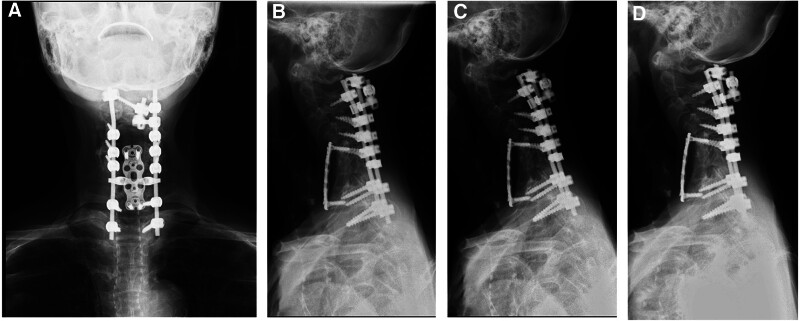
Cervical spine X-ray of the final observation. (A) The anteroposterior view; (B) the lateral view of the neutral posture; (C) flexion; (D) extension.

A 61-year-old female with chronic kidney disease undergoing hemodialysis presented with destructive spondyloarthropathy affecting the C6 and C7 (Fig. 1A–C). She presented with severe neck pain, paralysis, and numbness. Initial treatment involved a halo vest, followed by anterior cervical corpectomy and fusion (ACCF) spanning from C5 to Th1 (Fig. 2A and B). However, a week after the halo vest removal, a C5 fracture occurred, resulting in cervical kyphosis (Fig. 3A–C). Subsequent surgery involved posterior fusion from C2 to Th2 to address the kyphotic alignment (Fig. 4A and B). To prevent further kyphotic malalignment, we intended to perform rigid fixation on the proximal side; therefore, we placed a C2 laminar hook on the rightmost proximal side. 6 months later, the patient complained of headaches. Radiography revealed rod migration into the occipital bone, which prompted a third surgery to address the complications (Fig. 5A–C). The third surgery involved the partial shortening of the rod on the cranial side. Following this, the patient became asymptomatic and remained free of complications (Fig. 6A–D). At the last follow-up, 5 years after the third surgery, there were no changes in spinal instrument dislocation or alignment, and no recurrence of neck pain.

## 3. Discussion

The present case of occipital bone erosion following multistage posterior cervical spine surgery for DSA underscores the intricate challenges associated with managing such complex cases. Several key aspects were illuminated through the lens of this case, including the implications for surgical decision-making, complication avoidance, and postoperative monitoring. The indications for the initial surgery are debatable; however, the complete prevention of surgical complications in DSA is difficult, and it is important to know the types of complications and understand the mechanisms and responses to each complication. In the present case, the instrumentation loosened after surgery, creating rod-induced occipital bone erosion. This preventive mechanism has also been discussed.

### 3.1. Extent of fixation and rod protrusion

A significant lesson from this case is the importance of carefully determining the extent of fixation during initial treatment with DSA. In the present case, the vertebral body was highly collapsed, and the instability was severe. Therefore, A 360-degree fixation might have been better. Berta et al^[4]^ reported satisfactory results with simultaneous ACCF and posterior fixation.

Nakao et al^[5]^ emphasized the need to prevent rod migration and related complications by maintaining the rod protrusion under 2 mm during surgical procedures. This recommendation aligns with the observations in our case, in which rod migration into the occipital bone led to subsequent complications. Tailoring the extent of fixation and closely monitoring the rod position may mitigate the risk of such complications. Precise control of rod protrusion, particularly in patients with DSA, could serve as a proactive measure to enhance long-term stability and reduce migration-related issues.

### 3.2. Preoperative assessment of occipital position

Kobets et al^[6]^ highlighted the value of preoperative flexion-extension radiographs for assessing the occipital position during extension. By evaluating the interaction between the occipital bone and the cervical spine in various positions, surgeons can gain insights into the potential challenges related to postoperative alignment and rod migration. Our case resonates with this notion, as the migration of the rod into the occipital bone could have been potentially identified or predicted through thorough preoperative assessments. Incorporating these measures in the preoperative planning phase could contribute to better surgical decision-making and anticipation of complications.

### 3.3. Compensatory range of motion and occipital migration

Postoperative dynamics of the cervical spine play a crucial role in the development of complications, as highlighted by Ikuta et al.^[7]^ After posterior cervical fusion, compensatory mechanisms may lead to an increased range of motion in the upper cervical spine, potentially contributing to occipital migration. The occurrence of this phenomenon in our case is noteworthy, because the patient’s history of multistage surgeries and previous alignment issues may have contributed to the observed rod migration. Understanding the biomechanical changes in the cervical spine following surgical intervention is essential for predicting and preventing complications.

### 3.4. Clinical implications and future directions

The multifaceted challenges and lessons learned from this case have several clinical implications. First, surgical strategies for patients with DSA should be meticulously planned considering individual patient characteristics, comorbidities, and potential alignment changes. Proactive shortening of the rods to account for potential alignment shifts, as proposed by Nakao et al, warrants further consideration. Second, preoperative assessment of the occipital position, in line with the recommendations of Kobets et al, can provide insights into the potential complications. Finally, recognizing the compensatory increase in range of motion reported by Ikuta et al is crucial for understanding postoperative dynamics and preventing migration-related issues.

In conclusion, this case of occipital bone erosion following multistage posterior cervical spine surgery in a patient with DSA highlights the complexities of managing such cases. The present case underscores the significance of precise fixation, preoperative assessment, and understanding postoperative biomechanics. Incorporating these lessons into clinical practice may improve patient outcomes, reduce complications, and enhance surgical decision-making in similar cases.

## 4. Limitation

This study’s limitation lies in basing its conclusions on a single case report, limiting its generalizability to all DSA patients due to the unique aspects of the patient’s condition. Additionally, the study doesn’t represent diverse patient demographics, potentially affecting surgical outcomes. Future research with a broader patient cohort is needed to validate the findings and understand DSA management strategies more comprehensively.

## 5. Conclusion

Here, we present a complex case of occipital bone erosion following multistage posterior cervical spine surgery for destructive spondyloarthropathy. This case underscores the challenges in managing DSA, particularly in patients with comorbidities such as chronic kidney dysfunction. Tailored fixation strategies and proactive measures to address potential alignment changes are crucial to optimize surgical outcomes and reduce postoperative complications. This report contributes to the existing knowledge on postoperative complications in cervical spine surgery and encourages a patient-centered approach for treatment planning.

## Author contributions

**Writing – original draft:** Hirohito Hirata, Masatsugu Tsukamoto, Takaomi Kobayashi, Tomohito Yoshihara, Yu Toda.

**Writing – review & editing:** Tadatsugu Morimoto, Masaaki Mawatari.

**Validation:** Masatsugu Tsukamoto, Takaomi Kobayashi.

**Visualization:** Takaomi Kobayashi.

**Supervision:** Masaaki Mawatari.
